# Supplementary material for: AFLAP: assembly-free linkage analysis pipeline using k-mers from genome sequencing data
Source: Genome Biol. 2021 Apr 21;22:115. doi: 10.1186/s13059-021-02326-x (PMC8061198; doi:10.1186/s13059-021-02326-x)
Supplement: Supplementary file 5 — Additional file 5. Review history. [file 13059_2021_2326_MOESM5_ESM.docx]

Review History

**First round of review**

**Reviewer 1**

**Were you able to assess all statistics in the manuscript, including the appropriateness of statistical tests used? yes**

**Comments to author:**

Dear Authors,

I enjoyed reading your manuscript titled “AFLAP: Assembly-Free Linkage Analysis Pipeline using k-mers from whole genome sequencing data”. The manuscript is very well written and easy to follow. The simulation analysis is extremely detailed and helps understand how different variables affect the placement accuracy of markers. The use of these markers to orient and order scaffolds shows one of the applications of having accurate markers. I have some suggestions/questions that I have outlined below. One broad comment I have is that although the evaluation is thorough, it’s not apparent why such an approach would be significantly better than the read mapping based approach - except for the improved runtime. I would encourage you to provide some explanation on this either in the background or discussion section in the manuscript.

Comments:

Page 4 Line 54: Authors mention the lack of high-quality assemblies as a shortcoming for deriving assembly/variant calling based markers. High-quality assembly can mean different things for different applications. An assembly can be highly contiguous but may have very low base-level accuracy or vice-versa. What specific aspect of the high quality assembly authors want to highlight here? Please be specific.

Page 5 Line 72: What about long read mapping and SNP calling? Some discussion about this would be helpful given the increasing acceptance of technologies such as ONT and Pacbio.

Page 5 Line 77: Why authors choose k=31 for further analysis? Does this need to be determined for different species based on k-mer spectrum plots? For more heterozygous species, smaller k-mers should capture more diversity thereby more markers.

Page 6 Line 93: What are count files? Are you referring to k-mer count files generated by Jellyfish here? If so, please consider making it explicit.

Page 6 Line 114: AFLAT consists of multiple steps that contribute to runtime. What is the step that that takes most time to run? Some discussion around that would be helpful.

Page 7 Line 118: Authors use Abyss assembler as a part of their pipeline to assemble k-mer markers into larger markers. However, the sentence criticizes the use of genome assembly. Please consider using “whole-genome assembly” instead of “genome assembly” in this sentence.

Page 16 Line 328:

Authors indicate that the assembled markers of size > 61 bp correspond to complex SVs such as indels. Do you have any validation to support this statement? One potential experiment can be using the parental assemblies of A.thaliana, and aligning them against each other to find locations of SVs. You can then check how many of the derived markers lie in the regions of differences.

Page 19 Line 400:

Authors mention the ability of AFLAT to generate robust genetic maps using only 61 bp sized markers. What happens if you don’t use markers of size > 61 bp? In other words, how much value is added in genetic map construction due to these larger markers?

Page 19 Line 409:

How common is it to observe MNPs and what’s the effect of those on conventional assembly/align/calling approaches? Please cite some references after this statement to back up this claim.

Page 21 Line 438: Authors discuss the lower limit of the coverage when the accuracy is good. However, what’s the upper limit of coverage after which the increase in accuracy is not significant after adding more data? Essentially, after what depth, you observe data to be redundant for genetic map construction?

Page 24 Line 536: Did you use paired-end information here while assembling markers? Or you used markers as single-end reads for Abyss and pairing didn’t add much information? Pairing might help in capturing more complex SVs in the larger markers.

Page 24 Line 544: This statement needs more explanation. Please consider expanding on it a bit. Also, if the coverage is very high, say 40x, do the thresholds for the number of observations change?

Page 28 Line 598: How did you generate paired reads? What were the read lengths, error rate, and insert size?

Page 28 Line 600: Authors used FreeBayes for variant calling. FreeBayes has been shown to have a large number of False Positive SNPs compared to other variant callers such as GATK or Deepvariant. Is there a specific reason authors chose FreeBayes for their analysis?

Page 29 Line 631: Authors show the Hi-C contact map, which is a great visual validation for the overall correctness of the assembly. Do you have any examples to show here where the orientation/ordering error in the original scaffolds was corrected with genetic maps derived AFLAT? That way, you could show the Hi-C contact map before and after the correction. If the scaffolds are not correct, you will see a lot of off-diagonal signals indicating errors.

Table1 and 2: Please make the caption for this table more elaborate to explain what different columns mean. For example, just by looking at the table or its caption, it’s not clear what does “Markers used” mean. Please make numbers right aligned.

What is the runtime reported here? Is it the wall-clock time or CPU time? If it’s wall-clock time, please consider reporting CPU time as it generates a fair comparison irrespective of the number of cores/threads used.

**Reviewer 2**

**Were you able to assess all statistics in the manuscript, including the appropriateness of statistical tests used? Yes**

**Comments to author:**

Overview:

Genetic maps are conventionally produced sequencing a family of individuals, aligning the resulting reads to a reference genome to detect variants to be used as markers, and finally constructing the genetic map with those markers. Here the authors present a pipeline for constructing the genetic map in a reference free manner. First single copy k-mers (which represent heterozygous sites) are detected from the sequencing data. The k-mers are then assembled to larger sequences to group the k-mers deriving from the same variant. The markers are then extracted from these assembled sequences. The experimental results show that the reference free approach produces as accurate genetic maps as the conventional approach but is significantly faster.

Suggestions for revision:

1) 31-mer is defined twice: first on page 5, line 77, and then on page 6, line 92.

2) Page 16, line 328: "Fragments larger than k+k-1 are likely to be complex variants such as insertions and deletions." However, as shown in Figure S4c and S4d deletions (regardless of length) produce fragments of length k+k-2. A similarly inaccurate sentence appear on page 25, line 536.

3) Page 20, line 418: "nine-linkage group" is repeated twice.

4) Page 20, line 433: construction accurate -> construction of accurate

5) Page 28, line 614: "UC Davis Genome Center computing cluster" is repeated twice.

6) Table 2 iv: The number of linkage groups is always 5 here even for the two genomes with 19 and 9 chromosomes, respectively. Is this an error?

**Authors Response**

**Point-by-point responses to the reviewers’ comments:**

Reviewer 1:

1.“it’s not apparent why such an approach would be significantly better than the read mapping based approach - except for the improved runtime. I would encourage you to provide some explanation on this either in the background or discussion section in the manuscript.”

*AFLAP is indeed faster than conventional pipelines even when a genome assembly is available; however, often a chromosome-scale assembly is not available. Therefore, it allows high density maps for non-model species. It also validates the quality of genome assemblies when they are generated and can lead to their improvement as shown by the B. lactucae example. In addition, AFLAP utilizes a variety of polymorphisms, not just SNPs. We have revised the text to emphasize these points (see line 64 to 67).*

2.“Page 4 Line 54: Authors mention the lack of high-quality assemblies as a shortcoming for deriving assembly/variant calling based markers. High-quality assembly can mean different things for different applications. An assembly can be highly contiguous but may have very low base-level accuracy or vice-versa. What specific aspect of the high quality assembly authors want to highlight here? Please be specific.”

*The revised text: “However, reference assemblies are not available for all species and may not be available for pertinent genotypes, even in well-studied species. In addition, a single reference assembly of a species does not capture all variation within a species. Therefore, some variants segregating in the gametes of a specific cross may not be accessible when a genome assembly is used to identify polymorphisms, particularly if neither parent was the reference genotype.”*

3.Page 5 Line 72: What about long read mapping and SNP calling? Some discussion about this would be helpful given the increasing acceptance of technologies such as ONT and Pacbio.

*AFLAP is most appropriate for use with high accuracy reads such as Illumina libraries. The high error rates of ONT and PacBio CLR would introduce a large amount of noise into the analysis. The cost of preparation and sequencing of HiFi libraries of segregating populations are currently prohibitive. We have added points to the discussion (from line 467) to cover this.*

4.Page 5 Line 77: Why authors choose k=31 for further analysis? Does this need to be determined for different species based on k-mer spectrum plots? For more heterozygous species, smaller k-mers should capture more diversity thereby more markers.

*We chose k=31 because of work done by others (e.g. Rahman et al. 2018, elife) where they report that 31 is the longest k-mer length that can be efficiently represented on 64- bit machines using Jellyfish. Other k-mer lengths could be used but 31-mers worked well so we did not investigate this variable further. The scripts on Github can be modified by users if different k-mer lengths are desired. Increasing k-mer length will increase the complexity, memory requirements and time of k-mer counting as the number of potential k-mers drastically increase. We have added details to the methods (line 539). Smaller k-mers will not capture more diversity, but it may produce more markers as the length of extension to neighboring markers, when assembled is reduced. For example, if k=21, variants would have to be within 20 bp of one another to be identified in the same marker. This is discussed on line 540.*

5.Page 6 Line 93: What are count files? Are you referring to k-mer count files generated by Jellyfish here? If so, please consider making it explicit.

*Line 104 Modified to: “Single copy 31-mers were identified by analyzing peaks contained within JELLYFISH count files.”*

6.Page 6 Line 114: AFLAT consists of multiple steps that contribute to runtime. What is the step that that takes most time to run? Some discussion around that would be helpful.

*Line 129 Inserted: “The longest stage of AFLAP is k-mer counting performed by JELLYFISH; however, the pipeline is deployed in such a way that previously calculated JELLYFISH results can be used if the k-mer size is not changed. This saves a large amount of time should the user wish to permute their analysis through down sampling, or the addition/exclusion of individuals from linkage analysis.”*

7.Page 7 Line 118: Authors use Abyss assembler as a part of their pipeline to assemble k-mer markers into larger markers. However, the sentence criticizes the use of genome assembly. Please consider using “whole-genome assembly” instead of “genome assembly” in this sentence.

*Line 134 Modified as suggested.*

8.Page 16 Line 328: Authors indicate that the assembled markers of size > 61 bp correspond to complex SVs such as indels. Do you have any validation to support this statement? One potential experiment can be using the parental assemblies of A. thaliana, and aligning them against each other to find locations of SVs. You can then check how many of the derived markers lie in the regions of differences.

*We aligned the 1,000 largest Col markers (ranging from 777 bp to 6.3 Kb) to both the Col and Ler genome assemblies. All but one could be aligned to Col, with 970 aligning 100% to the genome assembly. No Col marker aligned 100% to Ler demonstrating that these markers were unique to Col. This information has been added to the result (line 190), methods (line 567), and Additional File 3 (details line 684). Since these Col markers could not be found in Ler, it supports that the larger markers do correspond to complex variants between the two homozygous Arabidopsis genotypes, such as insertions or closely linked multi-nucleotide variants.*

9.Page 19 Line 400: Authors mention the ability of AFLAP to generate robust genetic maps using only 61 bp sized markers. What happens if you don’t use markers of size > 61 bp? In other words, how much value is added in genetic map construction due to these larger markers?

*The major added value of using markers equal to and greater than 61 bp is density of markers. Genetic maps can be produced exclusively with markers equal to 61 bp, or with makers greater than 61 bp. In our analysis both classes of markers are distributed throughout the genome. Our current scripts report how many markers are produced in each category. This gives the user an indication of how divergent the parents are from one another because markers greater than 61 bp indicate multi nucleotide polymorphisms or structural variation. We had originally discussed this in page 18.*

10.Page 19 Line 409: How common is it to observe MNPs and what’s the effect of those on conventional assembly/align/calling approaches? Please cite some references after this statement to back up this claim.

*From line 430; We have added citations and discuss how prevalent these markers were in the maps.*

*17.Kaplanis J, Akawi N, Gallone G, McRae JF, Prigmore E, Wright CF, et al. Exomewide assessment of the functional impact and pathogenicity of multinucleotide mutations. Genome Research. 2019;29(7):1047-56.*

*18.Sandmann S, de Graaf AO, Karimi M, van der Reijden BA, Hellström-Lindberg E, Jansen JH, et al. Evaluating Variant Calling Tools for Non-Matched Next-Generation Sequencing Data. Scientific Reports. 2017;7(1):43169.*

11.Page 21 Line 438: Authors discuss the lower limit of the coverage when the accuracy is good. However, what’s the upper limit of coverage after which the increase in accuracy is not significant after adding more data? Essentially, after what depth, you observe data to be redundant for genetic map construction?

*Line 459. We now precede this sentence stating that 5x coverage is adequate for high confidence marker placement. Table 2 shows that map quality plateaus after running with 5x coverage, reaching 98% to 100% accuracy.*

12.Page 24 Line 536: Did you use paired-end information here while assembling markers? Or you used markers as single-end reads for Abyss and pairing didn’t add much information? Pairing might help in capturing more complex SVs in the larger markers.

*We assembled markers as single end reads. By assembling the k-mers unique to either haplotype (F1) or parent (F2 or RIL) we know the precise position of at least one variant in each assembled fragment. This enables rapid genotyping of the progeny using a single k-mer length for every individual. In addition, using single end assembly provided an excess of markers. This has been further explained in the methods (from line 559).*

13.Page 24 Line 544: This statement needs more explanation. Please consider expanding on it a bit. Also, if the coverage is very high, say 40x, do the thresholds for the number of observations change?

*We had variable coverage for Bremia progeny (from 5x to >50x) and did not encounter problems due to high coverage. It is likely that uniquely amplified errors in a library will be filtered by application of segregation distortion boundaries. In other words, uniquely amplified errors will not be common in a high enough proportion of the libraries to impact the analysis. This information has been inserted into the methods section (from line 576).*

14.Page 28 Line 598: How did you generate paired reads? What were the read lengths, error rate, and insert size?

*Line 627 and 635.* *These paired end reads were the same simulations as described in the preceding paragraph, generated using randomnreads.sh (BBMAP), and are detailed on the GitHub repository https://github.com/kfletcher88/CrossSimulator (noted in the manuscript). The error rate and insert size was left as default.*

15.Page 28 Line 600: Authors used FreeBayes for variant calling. FreeBayes has been shown to have a large number of False Positive SNPs compared to other variant callers such as GATK or Deepvariant. Is there a specific reason authors chose FreeBayes for their analysis.

*FreeBayes is a commonly used variant caller and is regularly used in the lab. We have not had issues with false calls.*

16.Page 29 Line 631: Authors show the Hi-C contact map, which is a great visual validation for the overall correctness of the assembly. Do you have any examples to show here where the orientation/ordering error in the original scaffolds was corrected with genetic maps derived AFLAP? That way, you could show the Hi-C contact map before and after the correction. If the scaffolds are not correct, you will see a lot of off diagonal signals indicating errors.

*Hi-C on the previous assembly version confirmed that there were errors in that assembly, which were corrected after re-orientation based on the AFLAP map. The image (sent directly to the editor) indicates that scaffold SHOA01000004.1 (indicated by red bar) contains two centromere-like signals (crosses off the diagonal). Reorienting with the AFLAP map broke this scaffold and assigned the centromere signatures to separate linkage groups (LG2 and LG11). This reordering is clearly shown in figures 6 b and c.*

17.Table1 and 2: Please make the caption for this table more elaborate to explain what different columns mean. For example, just by looking at the table or its caption, it’s not clear what does “Markers used” mean. Please make numbers right aligned.

What is the runtime reported here? Is it the wall-clock time or CPU time? If it’s wallclock time, please consider reporting CPU time as it generates a fair comparison irrespective of the number of cores/threads used.

*We have made the column headings more detailed and right-aligned numbers as requested by the reviewer. The time reported is wall-clock. Unfortunately, the scratch space this data was generated on was recently replaced, so we would need to rerun all simulations to obtain the CPU time. We appreciate the reviewer pointing this out and will be sure to report CPU time in future studies.*

Reviewer 2:

1) 31-mer is defined twice: first on page 5, line 77, and then on page 6, line 92.

Line 102; corrected, thanks.

2) Page 16, line 328: "Fragments larger than k+k-1 are likely to be complex variants such as insertions and deletions." However, as shown in Figure S4c and S4d deletions (regardless of length) produce fragments of length k+k-2. A similarly inaccurate sentence appear on page 25, line 536.

*Line 347 and 565; corrected. Thanks.*

3) Page 20, line 418: "nine-linkage group" is repeated twice.

*Line 440; corrected*

4) Page 20, line 433: construction accurate -> construction of accurate

*Line 454; corrected.*

5) Page 28, line 614: "UC Davis Genome Center computing cluster" is repeated twice.

*Line 650; corrected.*

6) Table 2 iv: The number of linkage groups is always 5 here even for the two genomes with 19 and 9 chromosomes, respectively. Is this an error?

*Yes, thank you for pointing this out*

**Second round of review**

**Reviewer 1**

I am happy with the authors' response to my comments.
